# *MST1R* Gene Variants Predispose Individuals to Tetralogy of Fallot

**DOI:** 10.1007/s43657-024-00175-9

**Published:** 2025-01-21

**Authors:** Zhiyu Feng, Xianghui Huang, Yuan Gao, Han Gao, Weilan Na, Chaozhong Tan, Shaojie Min, Yuquan Lu, Quannan Zhuang, Siyi Lin, Xiaojing Ma, Weicheng Chen, Weili Yan, Wei Sheng, Guoying Huang

**Affiliations:** 1 Children’s Hospital of Fudan University, 399 Wanyuan Road, Shanghai, 201002 China; 2 Shanghai Key Laboratory of Birth Defects, Shanghai, 201002 China; 3 Fujian Key Laboratory of Neonatal Diseases, Xiamen Children’s Hospital, Fujian, 361006 China; 4 Research Unit of Early Intervention of Genetically Related Childhood Cardiovascular Diseases (2018RU002), Chinese Academy of Medical Sciences, Beijing, 100730 China

**Keywords:** Congenital Heart Disease, Tetralogy of Fallot, Macrophage Stimulating 1 Receptor, Variants

## Abstract

Tetralogy of Fallot (TOF) is the most common cyanotic congenital heart malformation. While a few susceptibility genes for TOF have been identified, research on the genetic basis of TOF is limited. The *Macrophage stimulating 1 receptor* (*MST1R*) gene encodes the macrophage-stimulating protein receptor with tyrosine phosphatase activity that is involved in immune defense. In this study, we performed whole-exome sequencing (WES) on 10 TOF families and 50 sporadic TOF patients and identified a recessive homozygous missense mutation in *MST1R*, c.T2009G: p.V670G, in two offspring with TOF in a single family. Targeted sequencing of the *MST1R* gene showed enrichment for rare variants in 417 TOF patients compared with East Asians in Genome Aggregation Database Version 2 (gnomADv2_EAS). *MST1R*-deficient human induced pluripotent stem cells (hiPSCs) maintained normal pluripotency but differentiated into non-functional cardiomyocytes (CMs). Taken together, our findings indicate that *MST1R* may play a critical role in cardiac differentiation and genetic variations in *MST1R* may be associated with the pathogenesis of TOF.

**Supplementary Information:**

The online version contains supplementary material available at 10.1007/s43657-024-00175-9.

## Introduction

Tetralogy of Fallot (TOF) is a complex congenital cardiac malformation that is characterized by pulmonary stenosis, overriding aorta, ventricular septal defect (VSD), and right ventricular hypertrophy, leading to hemodynamic changes such as right ventricular hypertension and hypertrophy (Althali and Hentges 2022; Kuribayashi et al. 1990; Zhang et al. 2021). TOF is one of the most prevalent cyanotic congenital heart diseases (CHDs), with an incidence of 3–4 of 10,000 newborns (van der Linde et al. 2011). The main treatment for TOF is surgery. While the survival rates for TOF patients who undergo surgery are high, the corrective procedure often leaves residual anomalies that affect patient health and quality of life (O’Byrne et al. 2020).

Genetic factors play a significant role in the etiology of TOF. Approximately 20% of TOF patients are diagnosed with genetic syndromes, such as the 22q11.2 deletion syndrome (Morgenthau and Frishman 2018; Silversides et al. 2012). The genetic basis of non-syndromic TOF is particularly complex and involves the interaction of multiple genes and possible environmental factors; however, in many cases, the specific etiology is still unclear (Page et al. 2019). Few single gene defects that are associated with non-syndromic TOF have been identified (Reuter et al. 2019). *Fms Related Receptor Tyrosine Kinase 4* (*FLT4*) and *Notch Receptor 1* (*NOTCH1*) were implicated in non-syndromic TOF pathogenesis, and variants in *FLT4* and *NOTCH1* were found in 7% individuals with TOF (Page et al. 2019). Damaging and loss-of-function variants in the Vascular Endothelial Growth Factor (VEGF) pathway genes *Kinase Insert Domain Receptor* (*KDR*), *FLT4* and *IQ Motif Containing GTPase Activating Protein 1* (*IQGAP1*) have also been identified in TOF families (Reuter et al. 2019).

*Macrophage stimulating 1 receptor* (*MST1R*), also known as RON, is the tyrosine kinase receptor for macrophage-stimulating factor protein. Previous studies suggested that Ron signaling is crucial in maintaining embryonic survival during the peri-implantation stage, and complete ablation of Ron results in embryonic lethality in mice (Muraoka et al. 1999). Aberrant expression of MST1R has been implicated in the pathogenesis of epithelial tumors (Wang et al. 2007; Yao et al. 2013). Gene-based association tests and action-machine learning models identified *MST1R* as a significant genetic contributor to coronary artery disease (Hariharan and Dupuis 2021; Shapiro et al. 2023). The largest-scale genetic study of a single CHD cohort performed thus far, involving 2871 cases, using whole exome sequencing (WES) identified six loss-of-function variants in *MST1R* in six patients (four patients with left ventricular outflow tract obstruction, and the other two patients with CHD) (Jin et al. 2017). No study has reported the role of *MST1R* in TOF.

In this study, we conducted WES on 10 TOF families and 50 sporadic TOF patients and identified a recessive homozygous missense variant of *MST1R*. Targeted sequencing in 417 sporadic individuals with TOF revealed a significant relation of *MST1R* and the occurrence of TOF (Fisher exact test *p* = 0.0005). We generated *MST1R*-deficient human induced pluripotent stem cells (hiPSCs) and found that *MST1R* knockout (KO) hiPSCs were unable to differentiate into normal cardiomyocytes (CMs). Our results expand the understanding of the genetic variants linked to TOF and reveal a potential new role for *MST1R* in cardiac differentiation.

## Materials and Methods

### Study Subjects

Children presenting with TOF at the Children's Hospital of Fudan University from May 2015 to December 2020 were enrolled in this study. This study was conducted in alignment with the 2013 revised Declaration of Helsinki and was approved by the Ethics Committee of the Children's Hospital of Fudan University (approval code No. 2016121). Written informed consent was obtained from the parents or legal guardians of all participating children.

### WES and Targeted Sequencing

Genomic DNA was extracted from peripheral blood samples of the patients and their parents using the QIAamp DNA Blood Mini Kit (Qiagen, Germany), following the manufacturer's guidelines. WES was performed on the probands and their parents by Gemple Biotech Co. Ltd in Shanghai, China. For population allele frequency assessment and pathogenicity prediction, the frequencies of alleles in the population were obtained from the East Asians in Genome Aggregation Database Version 2 (gnomADv2_EAS), accessible at https://gnomad.broadinstitute.org/. In the trio analysis, we first classified variants according to the inheritance pattern. And then filtered out rare and deleterious mutations. (1) loss-of-function variants; (2) missense mutations: absence or minor allele frequency ≤ 0.01% in gnomADv2_exome_EAS and Combined Annotation Dependent Depletion (CADD) score > 20. Homozygous MAF would be especially calculated if the variants fit the recessive inheritance model. In the targeted sequencing, the *MST1R* gene was sequenced to an average depth of 100×; the mutation filtering for this targeted sequencing was carried out using the same criteria that were applied in the WES analysis.

### Gene-specific Variant Burden Analysis

Reference control data were sourced from gnomADv2, encompassing exome sequencing data derived from 251,494 individuals without known familial ties. In acknowledgment of ethnic specificity, our selection was confined to East Asian populations (*n* = 19,960) within gnomADv2 to serve as the control group. We downloaded the *MST1R* variants information from http://gnomad-sg.org/ and uploaded the excel including all *MST1R* variants in east Asian populations to https://cadd.gs.washington.edu/snv to get the CADD score which can quantify the pathogenicity of variations. the identified *MST1R* mutations in database were according to the same filtering criteria as the analysis on WES.

### AI Predictions

We initially attempted to model the structure of *MST1R* using the SWISS-MODEL servers. However, the generated models were deemed unsatisfactory because of the low homology with the template proteins. We then used AlphaFold, the advanced deep learning algorithm developed by DeepMind, for predicting the MST1R model. This approach aimed to obtain more stable structures of the wild-type (WT) MST1R protein.

### Immunohistochemistry (IHC)

The right ventricular outflow tract tissues from patients with TOF and controls were fixed in 10% neutral buffered formalin for approximately 48 h at room temperature. The fixed tissues were embedded in paraffin and sliced into sections with a thickness of 4 µM. IHC was performed using a IHC secondary antibody kit (Absin, Abs996) following the manufacturer's instructions. Briefly, the tissue sections were incubated with a primary antibody against MST1R at a dilution of 1:100 (Santa Cruz, sc-374,626) at room temperature for 30 min. The sections were then incubated with HRP-conjugated anti-rabbit/anti-mouse IgG antibodies for 30 min at room temperature. Sections were stained with DAB and hematoxylin for 5 min. MST1R expression was observed using a light microscope (Leica GmbH).

### hiPSC Culture and Differentiation

hiPSCs were grown on plates coated with Matrigel (Corning, 356,255) using mTeSR™ plus medium (Stemcell Technologies, 100–0276). When the cells covered approximately 90% of the plate, they were split using Accutase (Sigma, A6964) at a ratio of 1:5. hiPSCs were differentiated into beating CMs using a previously described method (Lian et al. 2012). In brief, cells at ∼ 80% confluence were cultured in RPMI 1640 (Thermo, 11,875,119) with B-27 minus insulin (Thermo, A1895601) containing 7 μm CHIR-99,021 (Selleck, S1263) to activate WNT signaling for two days. After two days, the medium was switched to RPMI 1640/B-27 minus insulin for 24 h. The cells were then cultured in fresh medium containing 5 μm IWR-1 (Selleck, S7086) for two days . From day 5 onwards, the cells were maintained in RPMI 1640 medium with B-27 supplement (Thermo, A245032), with medium changes occurring every other day. Beating CMs were observed around day 7–8 post-differentiation.

### Generation of *MST1R*-KO hiPSC Cell Lines

*MST1R*-deficienct hiPSC cell lines were created using the episomal vector–based CRISPR/Cas9 (Epi-CRISPR) genome editing system. Guide RNA (gRNA) was designed using DeepHF (https://www.deephf.com/) and cloned into the epiCRISPR vector (a kind gift by Dr Yongming Wang). EpiCRISPR-gRNA was transfected into hiPSCs by Lonza 4D Nucleofector (Lonza, AAF-1002X). *MST1R*-null clones were selected using puromycin (Thermo, A1113803) for one week and then screened by genomic sequencing and western blot.

### Reverse Transcription–quantitative Real-time Polymerase Chain Reaction (RT-qPCR)

Total RNA was extracted using TRIZOL reagent (Thermo, 15,596,018). RNA (1 µg) was reverse-transcribed into complementary DNA (cDNA) using the PrimeScript™ RT reagent Kit (Takara, RR036A). RT-qPCR was performed using the TB Green® Premix Ex Taq™ (Takara, RR420A) on a QuantStudio System (Thermo Fisher Scientific) following the manufacturers’ instructions. Primers are listed in Table S3.

### Western Blot

Total protein was extracted using RIPA lysis buffer (Thermo, 89,901) containing protease inhibitor cocktail (Thermo, 78,442). Protein samples (20 µg) were separated by 10% SDS-PAGE (Epizyme, PG112) and transferred to Polyvinylidene Fluoride (PVDF) membranes (Millipore, IPVH00010). The membranes were blocked with 5% non-fat milk in TBST (Beyotime, ST673) at room temperature for 1 h and then incubated overnight at 4 °C with primary antibodies against MST1R (Santa Cruz, sc-374,626) at 1:500 and Glyceraldehyde-3-Phosphate Dehydrogenase (GAPDH) (Proteintech, 10494-1-AP) at 1:10000. Membranes were washed with TBST three times for 10 min followed by incubation in secondary antibodies (CST, 7074/7076) at 1:3000 for 2 h at room temperature. The bands were visualized using the ChemiDoc Imaging System (Biorad XRS+).

### Alkaline Phosphatase (AP) Staining

hiPSCs were plated on 15 mm dishes coated with Matrigel and cultured for two to three days. AP staining was performed using the Quantitative Alkaline Phosphatase ES Characterization Kit (Millipore, SCR004) following the manufacturer’s instructions.

### Immunofluorescence Staining

To assess pluripotency, hiPSCs were digested into single cell suspensions using Accutase and then passaged at a 1:10 ratio onto 15 mm dishes coated with Matrigel. The cells were rinsed with PBS, followed by fixation with 4% paraformaldehyde for 15 min and permeabilization in 0.02% Triton-X100 for 15 min. Cells were washed in PBS three times for 5 min each. The cells were incubated with primary antibodies against Octamer-binding protein 4 (OCT4) (Abcam, ab181557) at 1:500 and Stage-Specific Embryonic Antigen 4 (SSEA4) (Abcam, ab16287) at 1:200 overnight at 4 °C. Cells were then incubated with Alexa Fluor conjugated secondary antibodies (Thermo, A32731 and A32723) at room temperature for 1 h. Cells were counterstained with DAPI (Millipore, MBD0015) at room temperature for 15 min. To assess differentiated CMs, cells were digested into single cell suspensions using TrypLE (Thermo, 12,604,021) and passaged at a 1:20 ratio onto 15 mm dishes coated with Matrigel. Cells were stained with antibodies against Troponin T2 (TNNT2) (Thermo, MA5-12960) Actinin Alpha 2 (ACTN2) (Proteintech, 14221-1-AP). Stained cells were observed under a Leica DMi8 fluorescence microscope.

### Transmission Electron Microscopy (TEM)

CMs were fixed with 2.5% glutaraldehyde solution at room temperature for 15 min and sectioned. Samples were analyzed using a transmission electron microscope (HITACHI).

### RNA Sequencing and Analysis

RNA-seq library preparation was conducted in accordance with the guidelines provided by the manufacturer (Vazyme, TR503-01). Libraries were subjected to high-throughput sequencing by the Haplox company using Illumina NoxaSeq 6000 platform (Jiangxi, China) with three biological replicates. The FASTQ data underwent adapter trimming using trim_galore (v0.6.4) and reads were mapped to the human genome reference (UCSC hg19) using Tophat software (Trapnell et al. 2009). The Cufflinks program (v2.2.1) was applied to allocate the mapped reads to human transcripts (UCSC hg19) using default settings. This process was to determine gene expression levels, which are represented by FPKM. Differentially expressed genes were determined using DESeq2 (Love et al. 2014) and defined by more than 1.5-fold change in FPKM and a *p*-value below 0.05.

### Statistical Analysis

Data were presented as mean ± standard deviation (SD), at least three independent experiments. Statistical analyses utilized Student’s t-test and two-tailed Fisher’s exact test. A *p*-value less than 0.05 was considered statistically significant. Figures were generated using GraphPad Prism version 9.0 (GraphPad Software).

### Web Sources

**UCSC Genome Browser**, https://genome.ucsc.edu.

CADD, https://cadd.gs.washington.edu/.

NCBI, http://www.ncbi.nlm.nih.gov/.

gnomAD, http://www.gnomad-sg.org/.

Life Design, http://www.deephf.com.

single nucleotide polymorphism database (dbSNP), https://www.ncbi.nlm.nih.gov/snp/.

## Results

### Identification of a *MST1R* Homozygous Mutation in a TOF Pedigree

**Fig. 1:**
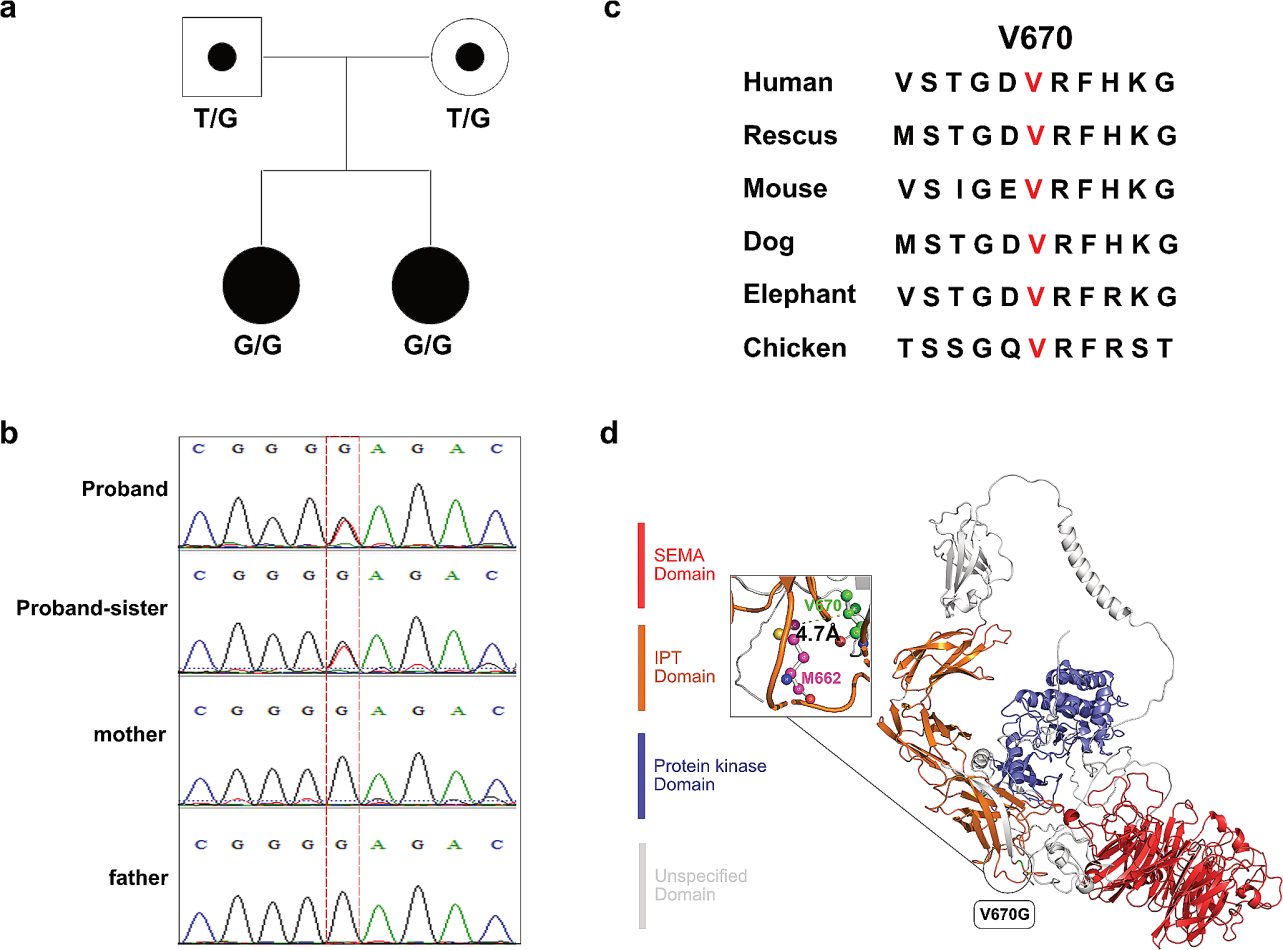
Homozygous mutation in *MST1R* identified in a TOF family. (**a**) Pedigree of the family with two TOF probands carrying a homozygous missense mutation (c.2009T > G) in the *MST1R* gene. (**b**) Sanger sequencing results confirming a recessive homozygous missense mutation in the two probands; their patients were heterozygous carriers. (**c**) Evolutionary conservation of V670 in the MST1R protein in several species. (**d**) Prediction of the 3D structures of the MST1R protein using AlphaFold. The location of the V670G variant is indicated (black circle, green segment). The hydrophobic interaction between V670 (green) and M662 (magenta, bold text) residue is indicated (black rectangle)

To explore possible genetic variants in TOF, we first performed WES in 10 TOF families and 50 sporadic TOF patients. We found that two TOF probands in one family carried the same homozygous missense mutation (NM_002447.4:c.2009T > G, dbSNP: rs201024956) in the *MST1R* gene, located at Val670, and this was confirmed by Sanger sequencing (Fig. 1a and b). Both of the heterozygous parents were healthy and had no cardiac defects. The population frequency of this variant is 0.005, and the mutation only occurred once in a homozygous form in gnomADv2_EAS (allele frequency 0.0001). The variant has not been reported previously in the ClinVar database, and can be classified as likely pathogenic based on specific criteria, including pathogenic moderate1 (PM1), pathogenic moderate2 (PM2), pathogenic Supporting 1 (PP1) and pathogenic Supporting 3 (PP3) according to the guidelines of the American College of Medical Genetics and Genomics (ACMG). Sequence analysis showed that p.Val670 was highly conserved among various species (Fig. 1c). To investigate the protein structure changes caused by the c.2009T > G mutation, we used AlphaFold to predict the MST1R protein structure. The distance between the beta carbon on valine at position 670 and the epsilon carbon of methionine at 688 position was close at 4.7 Å (< 5 Å), suggesting that hydrophobic interactions can be formed between these two residues (Fig. 1d).

### Reduced MST1R Protein Expression in TOF Patients

**Fig. 2:**
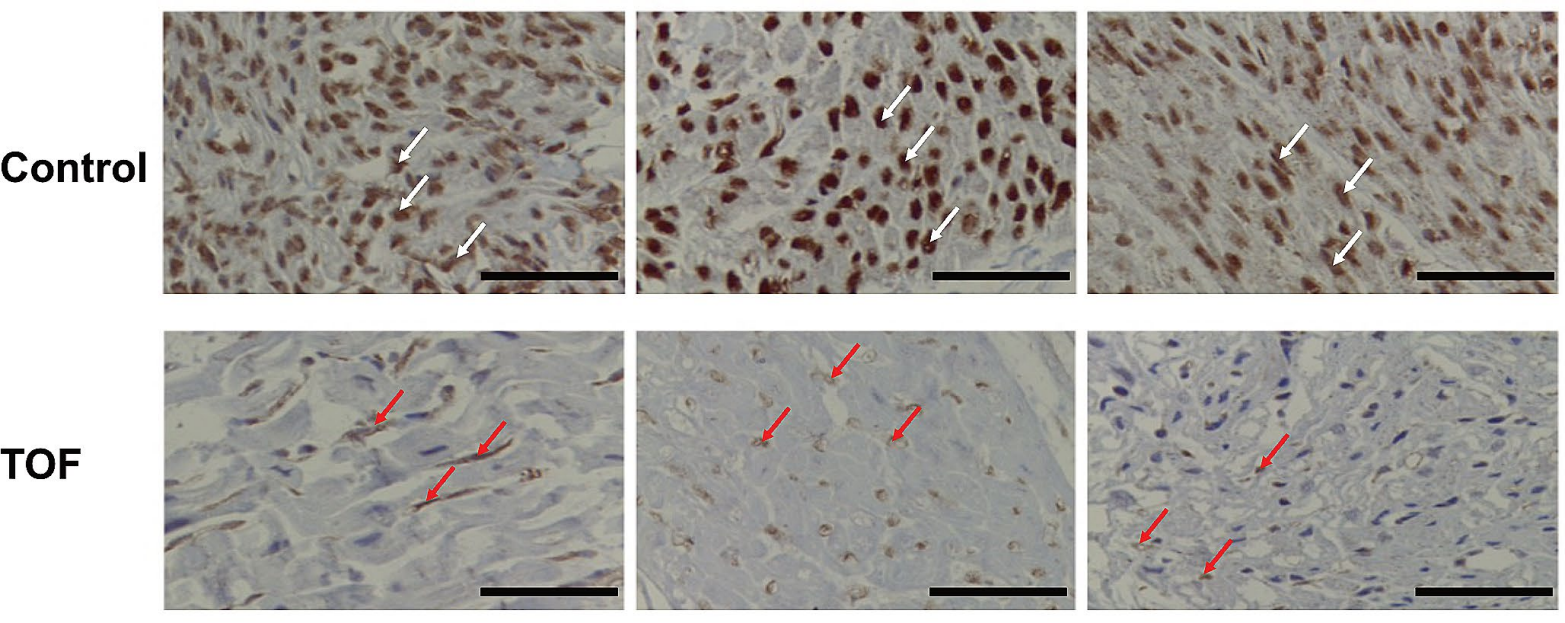
Decreased MST1R expression in cardiac tissues from TOF patients. Representative immunohistochemical staining images of MST1R in cardiac tissues from TOF patients and healthy controls. White arrow indicating MST1R in the Control, red arrow indicating MST1R in the TOF; Scale bars, 50 μm

We next used IHC to examine the expression of MST1R protein in cardiac tissue samples from TOF cases and healthy controls. The results showed that MST1R protein was markedly lower in the TOF samples compared with the controls (Fig. 2).

### Genetic Abnormalities in *MST1R* are Associated with TOF

**Fig. 3:**
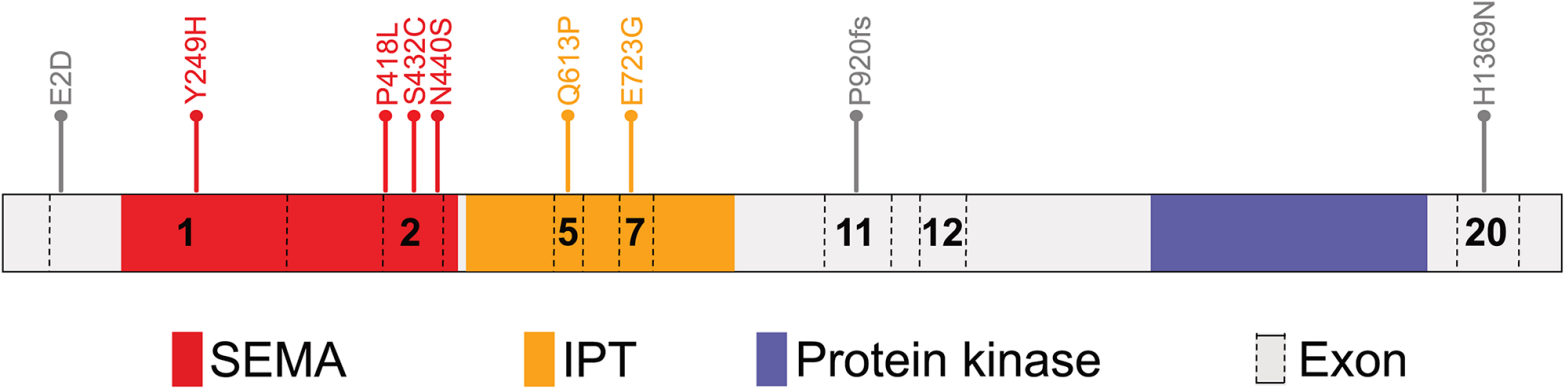
Distribution of rare variants in *MST1R*. Targeted sequencing of 417 individuals with TOF identified 9 *MST1R* variants. Schematic illustration of the MST1R protein and its domains and the location of identified variants is shown. Numbers indicate *MST1R* exons. SEMA, semaphorin domain; IPT, immunoglobulin-like plexin and transcription domain

We then performed targeted sequencing of *MST1R* in 417 TOF patients. The Clinical Profile Summary of 417 cases with TOF are summarized in Table S1. The results identified nine *MST1R* deleterious heterozygous variants that fulfilled the filtering criteria (a. loss-of-function variants; b. missense mutations: absence or minor allele frequency ≤ 0.01% in gnomADv2_exome_EAS and CADD score > 20) (Fig. 3, S1, Tables 1 and 2). Reference control data were sourced from gnomADv2_exom_EAS database, encompassing exome sequencing data derived from 19,960 unrelated individuals. In alignment with the procedures utilized for our TOF cohort, our screening process identified 50 rare pathogenic variants within the reference control population that meet our filtering criteria (Table S2). We compared the deleterious variants frequency of *MST1R* (9/834, 1.07%) in our study with those (50/19,960, 0.25%) in gnomADv2_exome_EAS database and found that the *MST1R* variants were associated with the occurrence of TOF (Fisher's exact test, OR: 5.492, 95% CI: 2.628–10.67, *p* = 0.0005) (Table 3).

**Table 1:** Genetic findings in TOF patients with MST1R variants

Patient ID	Variant^*^	Inheritance	Domain	MAF (exome_EAS)	CADD	Homozygous_MAF (exome_EAS)	ClinVar (Variation ID)	dbSNP (build 156)	ACMG classification	ACMG criteria
**WES family**										
1171	c.2009T > G: p.V670G	homozygous	IPT/TIG1	0.005	23.4	0.0001	NR	rs201024956	Likely pathogenic	PS1, PM2, PP1, PP3
1171_sister	c.2009T > G: p.V670G	homozygous	IPT/TIG1	0.005	23.4	0.0001	NR	rs201024956	Likely pathogenic	PS1, PM2, PP1, PP3
**Targeted sequencing**										
B273	c.6G > T: p.Glu2Asp	heterozygous	N/A	-	23.6	-	NR	Novel	VUS	PM2, PP2, PP3
B894	c.745T > C: p.Tyr249His	heterozygous	Sema	-	24.4	-	NR	Novel	Likely pathogenic	PM1, PM2, PP2, PP3
B247	c.1253 C > T: p.Pro418Leu	heterozygous	Sema	-	32	-	NR	Novel	Likely pathogenic	PM1, PM2, PP2, PP3
NO_0178	c.1294 A > T: p.Ser432Cys	heterozygous	Sema	-	23.2	-	NR	Novel	Likely pathogenic	PM1, PM2, PP2, PP3
NO_0265	c.1319 A > G: p.Asn440Ser	heterozygous	Sema	0	24.8	0	Benign (784,007)	rs2230592	VUS	PP3
A2076	c.1838 A > C: p.Gln613Pro	heterozygous	IPT/TIG1	0.00005	24.2	0	Benign (403,115)	rs35986685	VUS	PP3
NO_0357	c.2168 A > G: p.Glu723Gly	heterozygous	IPT/TIG2	-	21.2	-	NR	Novel	VUS	PM2, PP2, PP3
B684	c.2759delC: p.Pro920fs	heterozygous	N/A	-	18	-	NR	Novel	VUS	PM2, PM4, PP3
B111	c.4105 C > A:p.His1369Asn	heterozygous	N/A	-	6.248	-	NR	Novel	VUS	PM2, PP2
* GRCh37, NM_002447.4; MAF, minor allele frequency; CADD, combined annotation dependent depletion; N/A, no data available; NR, not reported; VUS, variant uncertain significance; PVS1, Pathogenic Very Strong 1; PM1, Pathogenic Moderate 1; PM2, Pathogenic Moderate 2; PM4, Pathogenic Moderate 5; PP1, Pathogenic Supporting 1; PP2, Pathogenic Supporting 2; PP3, Pathogenic Supporting 3; IPT/TIG, immunoglobulin, plexin, transcription factor-like/transcription factor immunoglobulin										

**Table 2:** Clinical features of TOF patients with MST1R variants

Patient ID	Gender	Age at first review (Days)	Clinical phenotype
B273	F	85	TOF
B894	M	234	TOF
B247	M	36	TOF, DA
NO_0178	M	150	TOF, DA
NO_0265	F	128	TOF
A2076	F	350	TOF
NO_0357	M	541	TOF, ASD
B684	M	371	TOF
B111	M	220	TOF

TOF, Tetralogy of Fallot; ASD, atrial septal defect; DA, double aortic arch

**Table 3:** Comparison of rare variants in sporadic TOF patients and healthy controls

	Allele count (identified MST1R mutations)	Allele count (no identified MST1R mutations)	*p*-value	OR	95% CI lower	95% CI upper
TOF patients	9	825	*0.0005*	5.492	2.628	10.67
Controls (gnomADv2_EAS)	50	19,910				

OR, odds ratio; CI, confidence interval; GnomADv2_EAS, East Asians in Genome Aggregation Database Version 2

### Generation of a *MST1R*-deficient hiPSC Line

**Fig. 4:**
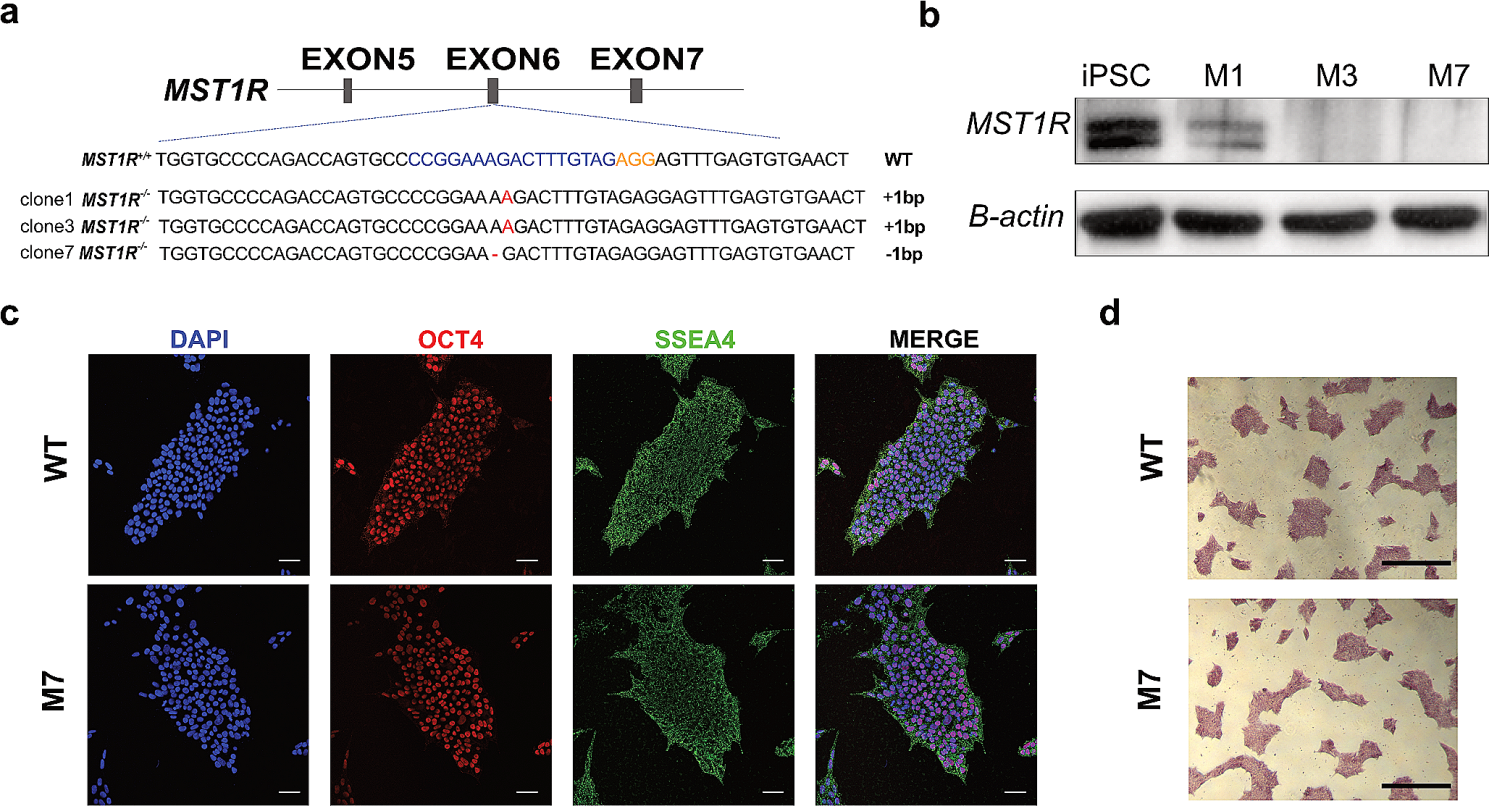
Generation and characterization of *MST1R*-deficient hiPSCs. (**a**) Schematic illustrating the generation of *MST1R* hiPSCs using gRNAs for exon 6 targeting. (**b**) Western blot analysis of WT and *MST1R*-deficient clones. (**c**) Representative immunofluorescence images of pluripotency markers OCT4 (red) and SSEA4 (green) in WT and M7 clones with DAPI (blue) staining. Scale Bars, 200 μm. (**d**) Alkaline phosphatase staining of WT and M7 clones. Scale bars, 200 mm

To examine the role of *MST1R* in cardiac development, we used the Epi-CRISPR gene-editing technology to generate *MST1R* KO hiPSCs for directed-CMs differentiation. The guide RNA (gRNA) was designed to target exon 6 of *MST1R* (Fig. 4a and Table S3). Positive clones were screened by Sanger sequencing and western blot; *MST1R* KO clone 7 was selected for subsequent experiments (Fig. 4b and S2). Deletion of *MST1R* did not disrupt the pluripotency of hiPSCs, as detected by immunofluorescence staining of pluripotency markers OCT4 and SSEA4 and alkaline phosphatase staining (Fig. 4c and d).

### Ablation of *MST1R* Impairs Human CM Differentiation

**Fig. 5:**
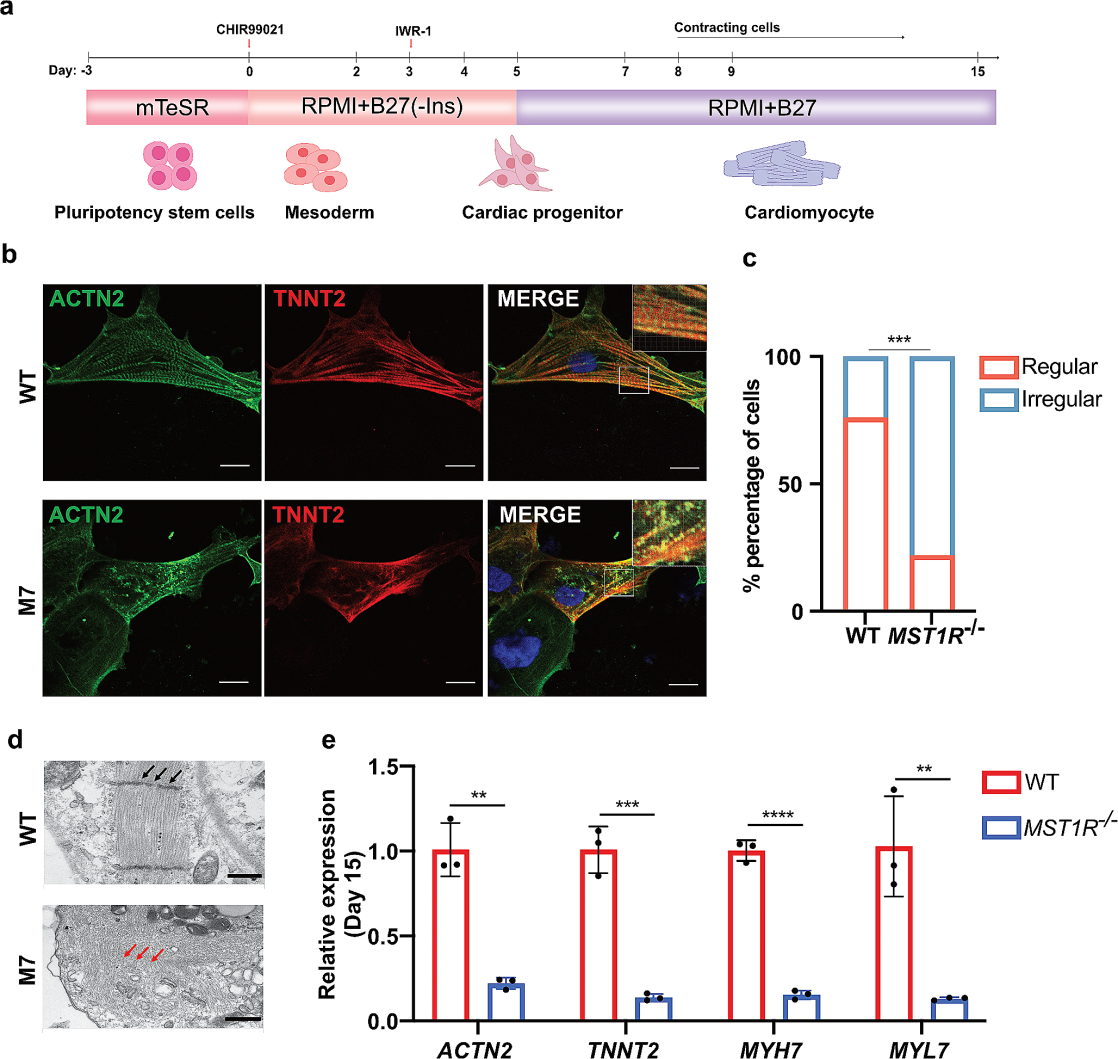
Phenotypic characterization of the *MST1R*-deficient CMs. (**a**) A schematic of the protocol for converting hiPSCs into CMs by modulating Wnt signaling activity. (**b**) Representative immunofluorescence images of WT-CMs and M7-CMs stained with ACTN2 (green), TNNT2 (red) and DAPI (blue). Scale Bars, 15 μm. (**c**) Quantification of irregularly organized sarcomeres in WT (*n* = 25) and *MST1R* KO (*n* = 27) day 15 differentiated cardiomyocytes. *n* = 25 (WT) and *n* = 27 (*MST1R* KO) biological independent samples. ***p < 0.001 by Fisher’s exact. (**d**) Transmission electron microscopy of day 15 differentiated cardiomyocytes. Scale Bars, 500 nm. (**e**) Expression level of cardiomyocytes structural genes (*ACTN2*, *MYH7* and *MYL7*) and contraction regulatory gene (*TNNT2*). *n* = 3, **p < 0.01, ***p < 0.001, ****p < 0.0001 by Student's *t* test

hiPSCs were differentiated into CMs using a 2D differentiation protocol as previously described (Fig. 5a) (Lian et al. 2012). Using the RNA-seq database maintained by our research group, we first conducted a cluster analysis of gene expression trends during CM differentiation (day 0–9) using Mfuzz tools. The results indicated an increase in MST1R expression on day 4, a critical period for cardiac differentiation (cardiac precursor cell stage). This pattern of expression is similar to that of key cardiac development genes such as *Notch Receptor 2* (*NOTCH2*) (Liu et al. 2015), *SMAD Family Member 4* (*SMAD4*) (Moskowitz et al. 2011), and *Potassium Voltage-Gated Channel Subfamily Q Member 1* (*KCNQ1*) genes (de la Rosa et al. 2013) (Fig. S3).

We then performed immunofluorescence staining of key sarcomere structure markers Actinin Alpha 2 (ACTN2) and Troponin T2 (TNNT2) in day 15 differentiated *MST1R* KO CMs. As shown in Fig. 5b and c, compared with wild-type CMs, *MST1R* KO CMs exhibited disordered sarcomeric organization, and there was a significant increase in the proportion of cells with abnormal sarcomeres (*p* < 0.001). We next used transmission electron microscopy (TEM) for visualization of sarcomere structures. We observed disorganized sarcomeres and disintegrated Z disc structures in day 15 differentiated *MST1R* KO CMs (Fig. 5d). Furthermore, the application of reverse transcription quantitative PCR (RT-qPCR) analysis revealed a marked decrease in the expression levels of several myofiber-specific structural and contraction regulatory genes, encompassing *ACTN2*, *Myosin Heavy Chain 7* (*MYH7*), *Myosin Light Chain 7* (*MYL7*) and *TNNT2* (Fig. 5e).

### Abnormal mRNA Transcript Levels of *MST1R* KO hiPSC-CMs

**Fig. 6:**
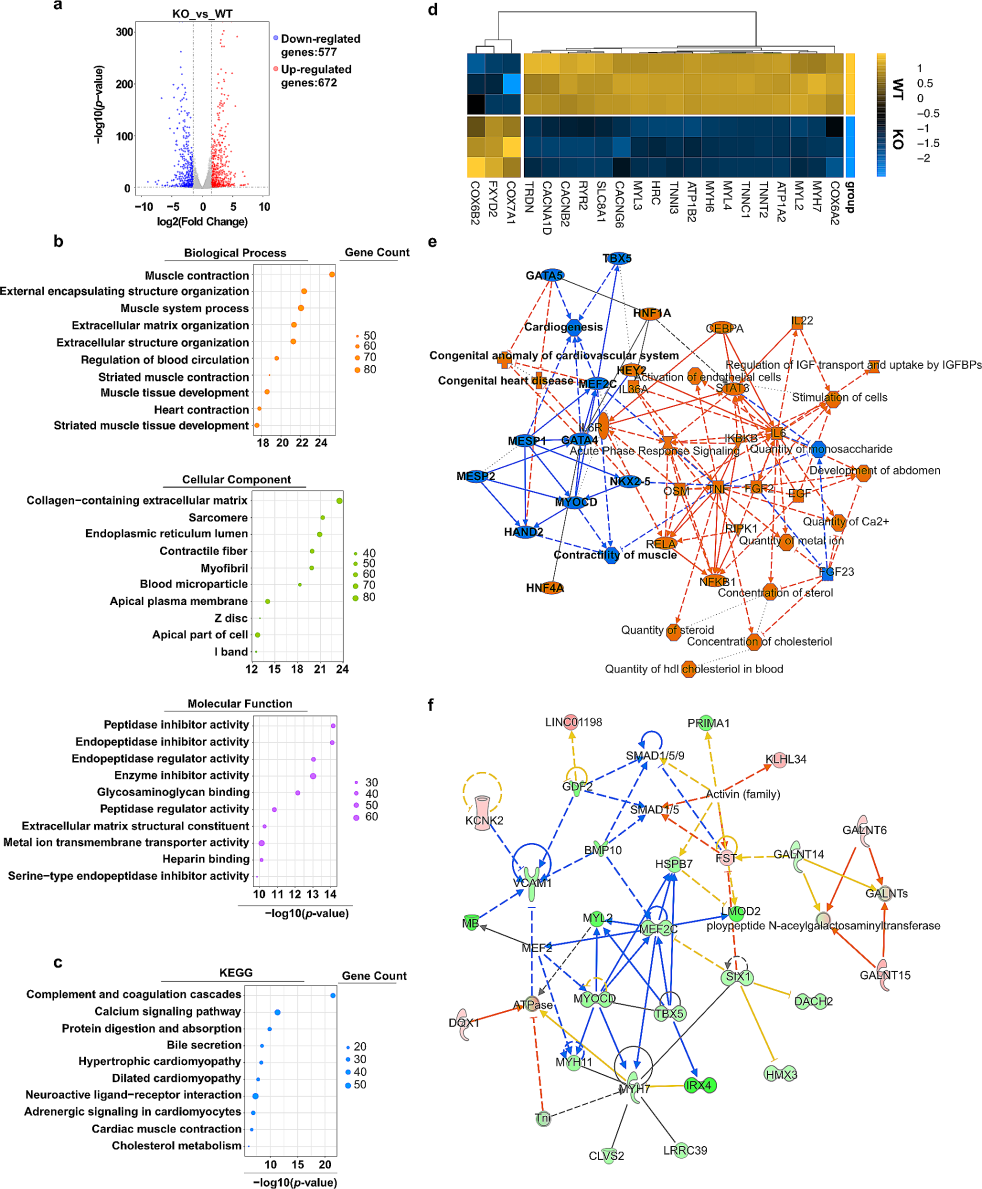
Gene expression analysis by RNA-seq of CMs derived from hiPSCs at day 15. (**a**) Volcano plot showing 672 up-regulated genes (red dots) and 577 down-regulated genes (blue dots). (**b**) GO enrichment analysis of differentially expressed genes in BP, CC and MF terms. (**c**) KEGG pathway analysis of differentially expressed genes. (**d**) Heatmap of differentially expressed genes associated with calcium signaling pathway and cardiac muscle contraction. (**e**) Representative IPA graphic summary of key biological themes. (**f**) The cardiovascular system development–related protein interaction network generated by IPA core analysis. Genes with increased expression are indicated in red; genes with decreased expression are indicated in green

To investigate the phenotypic characteristics of CMs derived from hiPSCs with *MST1R* gene knockout, we performed transcriptomic profiling and functional enrichment analyses with RNA-seq on hiPSC-CMs (day 15). The results identified 672 significantly up-regulated and 577 significantly down-regulated genes; a volcano plot is shown in Fig. 6a. Gene Ontology (GO) analysis indicated that the most significantly impacted biological processes terms included muscle contraction, muscle tissue development, and heart contraction (Fig. 6b). The terms "sarcomere", "contractile fiber", "myofibril" and "z disc" were significantly enriched in the cellular component category, and within the molecular function category, significant enrichment was observed for terms such as "metal ion transmembrane transporter activity". Kyoto Encyclopedia of Genes Genome (KEGG) analysis showed significant alterations in biological pathways; these changes pertained to pathways associated with the calcium signaling pathway, adrenergic signaling in cardiomyocytes and cardiac muscle contraction (Fig. 6c). A heatmap that delineates the differentially expressed genes within these impacted biological processes and pathways is shown in Fig. 6d. We further used Ingenuity Pathway Analysis (IPA) to generate networks based on interactions between these genes (Fig. 6e and f, S4 and S5). Overall, these findings imply that *MST1R* deficiency influences the cardiac differentiation of hiPSCs towards abnormal functioning.

## Discussion

TOF is the most common and severe cyanotic variant of CHD. The etiological underpinnings of non-syndromic TOF remain elusive, with few single gene variants identified as definitive causes of phenotypic and characteristics in TOF (Harold 2014; Page et al. 2019). Here, we provided the first genetic and functional evidence demonstrating that abnormalities in the *MST1R* gene predispose individuals to non-syndromic TOF.

The efficacy of WES in identifying mutations in individuals with genetic disorders is approximately 25%. Recent studies revealed that deleterious variants in the *NOTCH1* and *FLT4* genes are significantly implicated in the pathogenesis of TOF (Page et al. 2019; Yang et al. 2013). In this study, we performed WES on 10 families with TOF and 50 individuals with sporadic TOF and identified a recessive homozygous missense mutation in the *MST1R* gene, c.T2009G: p.V670G, in two siblings within one of the TOF-affected families. Previous studies using WES demonstrated that rare genetic variants in *MST1R* contribute to Lady Windermere syndrome and nasopharyngeal carcinoma (Becker et al. 2017; Dai et al. 2016). To evaluate the correlation between deleterious variants of the *MST1R* gene and TOF, we performed targeted exon sequencing of the *MST1R* gene in a cohort of 417 individuals with TOF. Upon comparison with the East Asian population in GnomADv2 database, a significant association was discovered between *MST1R* gene variations and TOF (*p*-value of 0.0005).

In previous studies, immunohistochemical analysis revealed the expression of wild-type MST1R in various cancerous and adjacent cancerous tissues, including pancreatic and prostate cancers (Batth et al. 2021; Li et al. 2019). In this study, IHC demonstrated that the expression of MST1R protein in the right ventricular outflow tract tissues was lower in patients with TOF compared with healthy subjects (Fig. 2). We were only able to present limited IHC results because of challenges in acquiring cardiac tissues, particularly from healthy control subjects.

iPSCs and their derivative CMs are an important resource for modeling and investigating heart malformations (Doyle et al. 2015; Kathiriya et al. 2021). Many advancements have been achieved through the use of hiPSCs-CMs models in characterizing CHDs such as atrial septal defect (ASD), VSD, and TOF (Ang et al. 2016; Grunert et al. 2020). Progresses in CRISPR technologies have significantly broadened the capabilities for DNA editing, enhancing both the efficiency and precision of these techniques (Musunuru 2022). Taken together, these developments hold considerable promise for applications in cardiac development. We used epiCRISPR, a novel CRISPR/Cas9 gene editing system, to achieve highly efficient gene knockouts in human pluripotent stem cells (hPSCs) and generating *MST1R* KO cell lines. The epiCRISPR system is distinguished by its capability to generate exceptionally high insertion/deletion (indel) rates, achieving efficiencies of up to 100% (Xie et al. 2017). The *MST1R* KO iPSCs were obtained without impacting their pluripotency and then differentiated into CMs (Fig. 4). Immunofluorescence analysis showed that approximately 75% of CMs with *MST1R* KO presented disorganized sarcomeric organization 15 days following CM differentiation. TEM assessments revealed that CMs with *MST1R* KO exhibited disrupted Z-disc structures (Fig. 5).

Current methods to create heart cells from stem cells produce a mix of different types of heart cells (Hemmi et al. 2014). In heart development, there are two main areas formed: the first heart field (FHF) and the second heart field (SHF), each marked by specific genes such as *ISL LIM Homeobox 1* (*Isl1*), *T-Box Transcription Factor 1* (*Tbx1*) and *Heart and Neural Crest Derivatives Expressed 2* (*Hand2*) (Buckingham et al. 2005; Cai et al. 2003; Jerome and Papaioannou 2001; Srivastava et al. 1997). However, attempts to specifically make heart cells from these fields using stem cells have not been very efficient, with about a 20% success rate. The process of making heart cells from human stem cells often ends up mixing heart cells from different areas and including unwanted cells. This issue has made it difficult to study how specific types of heart cells could be used in new heart disease treatments (Galdos et al. 2023; Yamaguchi et al. 2023). Zhang and colleagues developed a new technique using human stem cells engineered to report on two key heart genes to sort out heart cells similar to those from FHF and SHF, however, this approach faced some problems, like the method used to engineer the stem cells, low success in making specific types of heart cells, and contamination with other cell types (Zhang et al. 2019). Our IHC results showed impaired expression of MST1R in the SHF, but iPSCs were not able to be distinguished. Modeling TOF presents considerable challenges because of its impacts on inducing multiple malformations at the organ level. Therefore, heart organoids or other three-dimensional models will be potentially more suitable for investigating TOF functions in heart malformations.

To explore the mechanisms underlying the alterations in cardiac-specific differentiation driven by *MST1R* deficiency, transcriptomic profiles generated through RNA-seq were analyzed. The enriched pathways in the *MST1R* KO CMs were primarily related to myocardial development and contraction.

MST1R signaling is crucial for the proliferation, differentiation and migration of various cell types (Yao et al. 2013). In the context of myocardial development, *MST1R* might influence the proliferation and migration of CMs or cardiac progenitor cells, contributing to the formation of cardiac muscle tissue. The extracellular matrix (ECM) plays a critical role in transmitting contractile forces across the myocardium (Li et al. 2014). *MST1R* involved in cell-matrix interactions could suggest a role in modulating the ECM composition or adhesion of CMs to the ECM, impacting the efficiency of force transmission and overall cardiac contractility. *MST1R* activation triggers multiple intracellular signaling pathways, including Mitogen-Activated Protein Kinase (MAPK) and Phosphoinositide 3-Kinase/Protein Kinase B (PI3K/Akt) (Chakedis et al. 2016; Yao et al. 2013). By activating these signaling pathways, *MST1R* can influence the activity of various transcription factors to regulate the expression of downstream genes. Our RNA sequencing data revealed that CMs lacking MST1R showed different levels of expression in genes that are similar to those involved in heart muscle contraction, the sarcomere structure, and calcium signaling pathways. Examples of these genes include *TNNT2*, *Myosin Heavy Chain 6* (*MYH6*), *Myosin Light Chain 2* (*MYL2*), *Calcium Voltage-Gated Channel Auxiliary Subunit Gamma 6* (*CACNG6*), and *Calcium Voltage-Gated Channel Subunit Alpha1 D* (*CACNA1D*). Future studies will focus on understanding how *MST1R* exactly influences these genes during the development of the heart in embryos.

Our study offers novel insights into the genetic etiology of TOF. However, it’s important to acknowledge the inherent limitations that accompany our findings. The use of hiPSC models, while invaluable for elucidating gene function and modeling disease pathology, may not fully reveal the complex in vivo environment of cardiac development and disease. The differentiation efficiency and the precise recapitulation of the CM lineage from hiPSCs, especially regarding the SHF contribution, remain challenging and could affect the role of *MST1R* in cardiac morphogenesis.

## Conclusions

We are the first to identify the link between the *MST1R* gene and non-syndromic TOF. The combination of clinical evaluation, genetic analysis and cellular functional assays collectively suggest that *MST1R* may be involved in the pathogenesis of certain types of congenital heart disease.

## Electronic Supplementary Material

Below is the link to the electronic supplementary material.

Supplementary Material 1

## Data Availability

All data and material are available from the corresponding authors on reasonable request.

## Abbreviations

ACMG: American College of Medical Genetics and Genomics
ACTN2: Alpha actinin 2
AP: Alkaline phosphatase
ASD: Atrial septal defect
CADD: Combined Annotation Dependent Depletion
CHDs: Congenital heart diseases
CMs: Cardiomyocytes
DA: Double aortic arch
hiPSCs: Human induced pluripotent stem cells
IPA: Ingenuity pathway analysis
IPT/TIG: Immunoglobulin, plexin, transcription factor-like/transcription factor immunoglobulin
KEGG: Kyoto Encyclopedia of Genes Genome
MAF: Minor allele frequency
MST1R: Macrophage stimulating 1 receptor
NR: Not reported
N/A: No data available
TEM: Transmission electron microscopy
TNNT2: Troponin T2
TOF: Tetralogy of Fallot
VUS: Variant uncertain significance
WES: Whole exome sequencing
