# Supplementary material for: MST1R Gene Variants Predispose Individuals to Tetralogy of Fallot
Source: Phenomics. 2025 Jan 21;4(6):548–61. doi: 10.1007/s43657-024-00175-9 (PMC11889330; doi:10.1007/s43657-024-00175-9)
Supplement: Supplementary file 1 — Supplementary Material 1 [file 43657_2024_175_MOESM1_ESM.docx]

**Table S1 Statistics of sex and age of 417 TOF patients**

| **Phenotypes** | **Cases (number)** | **Sex ratio (M/F)** | **Median age at first visit (IQR), Days** |
| --- | --- | --- | --- |
| TOF | 417 | 1.45 | 237 (0-5237) |
| M/F, male/female; IQR, interquartile range | | | |

| **Table S2 Rare pathogenic variants from GnomADv2_exom_EAS database** | | | | | | | | |
| --- | --- | --- | --- | --- | --- | --- | --- | --- |
| **Chrom** | **Pos** | **Ref** | **Alt** | **RawScore** | **PHRED** | **Allele Count  East Asian** | **Allele Number  East Asian** | **MAF (East asian)** |
| 3 | 49924998 | G | C | 2.316128 | 22.1 | 1 | 18390 | 5.44E-05 |
| 3 | 49927390 | C | T | 4.668387 | 32 | 1 | 19950 | 5.01E-05 |
| 3 | 49927457 | G | C | 4.391302 | 32 | 1 | 18350 | 5.45E-05 |
| 3 | 49927985 | C | T | 4.171736 | 29.2 | 1 | 18390 | 5.44E-05 |
| 3 | 49928691 | T | C | 2.050652 | 20.2 | 1 | 18390 | 5.44E-05 |
| 3 | 49928845 | C | G | 4.449961 | 32 | 1 | 18388 | 5.44E-05 |
| 3 | 49928923 | C | G | 3.180627 | 24 | 1 | 18388 | 5.44E-05 |
| 3 | 49928926 | A | G | 2.325492 | 22.1 | 1 | 19948 | 5.01E-05 |
| 3 | 49928962 | A | G | 3.760176 | 26.1 | 1 | 18390 | 5.44E-05 |
| 3 | 49928978 | G | A | 7.797487 | 39 | 1 | 19948 | 5.01E-05 |
| 3 | 49929190 | C | T | 5.368743 | 33 | 1 | 19954 | 5.01E-05 |
| 3 | 49929191 | G | A | 4.769886 | 32 | 1 | 18394 | 5.44E-05 |
| 3 | 49929228 | GGCC TGGT | G | 4.790245 | 32 | 1 | 18394 | 5.44E-05 |
| 3 | 49932612 | C | G | 2.728333 | 23 | 1 | 18394 | 5.44E-05 |
| 3 | 49932639 | C | A | 6.143816 | 35 | 1 | 18394 | 5.44E-05 |
| 3 | 49932663 | C | T | 3.88088 | 26.8 | 1 | 18394 | 5.44E-05 |
| 3 | 49933315 | T | C | 4.947231 | 33 | 1 | 18208 | 5.49E-05 |
| 3 | 49933411 | C | A | 2.944842 | 23.4 | 1 | 18098 | 5.53E-05 |
| 3 | 49933704 | C | T | 2.509426 | 22.6 | 1 | 19954 | 5.01E-05 |
| 3 | 49933996 | C | T | 3.252775 | 24.2 | 1 | 19950 | 5.01E-05 |
| **Table S2 *Continued*** | | | | | | | | |
| **Chrom** | **Pos** | **Ref** | **Alt** | **RawScore** | **PHRED** | **Allele Count  East Asian** | **Allele Number  East Asian** | **MAF (East asian)** |
| 3 | 49934035 | G | C | 2.874742 | 23.3 | 1 | 18392 | 5.44E-05 |
| 3 | 49934159 | T | G | 2.090198 | 20.6 | 1 | 16704 | 5.99E-05 |
| 3 | 49934221 | C | T | 6.929387 | 36 | 1 | 13008 | 7.69E-05 |
| 3 | 49934309 | T | G | 2.265144 | 21.8 | 1 | 11172 | 8.95E-05 |
| 3 | 49935037 | G | T | 2.52137 | 22.6 | 1 | 19948 | 5.01E-05 |
| 3 | 49935039 | T | C | 2.381757 | 22.3 | 1 | 18390 | 5.44E-05 |
| 3 | 49935044 | G | A | 3.845029 | 26.5 | 1 | 18390 | 5.44E-05 |
| 3 | 49935110 | G | C | 2.270113 | 21.9 | 1 | 18356 | 5.45E-05 |
| 3 | 49935503 | T | A | 7.137914 | 37 | 1 | 18394 | 5.44E-05 |
| 3 | 49935515 | G | A | 2.267427 | 21.8 | 1 | 19950 | 5.01E-05 |
| 3 | 49935595 | C | T | 3.855154 | 26.6 | 1 | 18388 | 5.44E-05 |
| 3 | 49935614 | TG | T | 3.10498 | 23.8 | 1 | 18370 | 5.44E-05 |
| 3 | 49935616 | C | T | 3.74304 | 26 | 1 | 18370 | 5.44E-05 |
| 3 | 49935999 | C | A | 2.674723 | 22.9 | 1 | 18392 | 5.44E-05 |
| 3 | 49936304 | TC | T | 3.940792 | 27.2 | 1 | 18394 | 5.44E-05 |
| 3 | 49936351 | C | CT | 3.192856 | 24 | 1 | 18394 | 5.44E-05 |
| 3 | 49936507 | C | T | 5.768106 | 34 | 1 | 18394 | 5.44E-05 |
| 3 | 49936509 | T | C | 4.998368 | 33 | 1 | 18394 | 5.44E-05 |
| 3 | 49936624 | G | T | 3.690167 | 25.7 | 1 | 18394 | 5.44E-05 |
| 3 | 49939938 | A | G | 4.14393 | 29 | 1 | 18394 | 5.44E-05 |
| 3 | 49940118 | G | A | 2.840069 | 23.2 | 1 | 19922 | 5.02E-05 |
| 3 | 49940121 | GC | G | 2.089286 | 20.5 | 1 | 19918 | 5.02E-05 |
| **Table S2 *Continued*** | | | | | | | | |
| **Chrom** | **Pos** | **Ref** | **Alt** | **RawScore** | **PHRED** | **Allele Count  East Asian** | **Allele Number  East Asian** | **MAF (East asian)** |
| 3 | 49940208 | G | A | 3.894553 | 26.9 | 1 | 18386 | 5.44E-05 |
| 3 | 49940388 | T | C | 2.839775 | 23.2 | 1 | 18312 | 5.46E-05 |
| 3 | 49940391 | T | C | 2.149718 | 21 | 1 | 18314 | 5.46E-05 |
| 3 | 49940424 | C | T | 3.259857 | 24.2 | 1 | 18340 | 5.45E-05 |
| 3 | 49940501 | G | A | 3.628969 | 25.5 | 1 | 18248 | 5.48E-05 |
| 3 | 49940504 | C | T | 3.83259 | 26.5 | 1 | 18242 | 5.48E-05 |
| 3 | 49940619 | G | A | 4.213088 | 29.6 | 1 | 19768 | 5.06E-05 |
| 3 | 49940624 | T | G | 2.56599 | 22.7 | 1 | 18232 | 5.48E-05 |

**Table S3 gRNA used for *MST1R* knockout and primers used for RT-PCR analysis**

| *MST1R* guide | CCGGAAAGACTTTGTAGAGG |  |
| --- | --- | --- |
| RT-qPCR-GAPDH | GGAGCGAGATCCCTCCAAAAT | GGCTGTTGTCATACTTCTCATGG |
| RT-qPCR-ACTN2 | CGTCGCTGACAGAGGTGC | CACCGATCATTGACATTCACAGC |
| RT-qPCR-TNNT2 | AAGAAGAAGATTCTGGCTGAGAG | ACTTTCTGGTTATCGTTGATCCT |
| RT-qPCR-MYH7 | TCGTGCCTGATGACAAACAGGAGT | ATACTCGGTCTCGGCAGTGACTTT |
| RT-qPCR-MYL7 | ACATCATCACCCATGGAGACGAGA | GCAACAGAGTTTATTGAGGTGCCC |


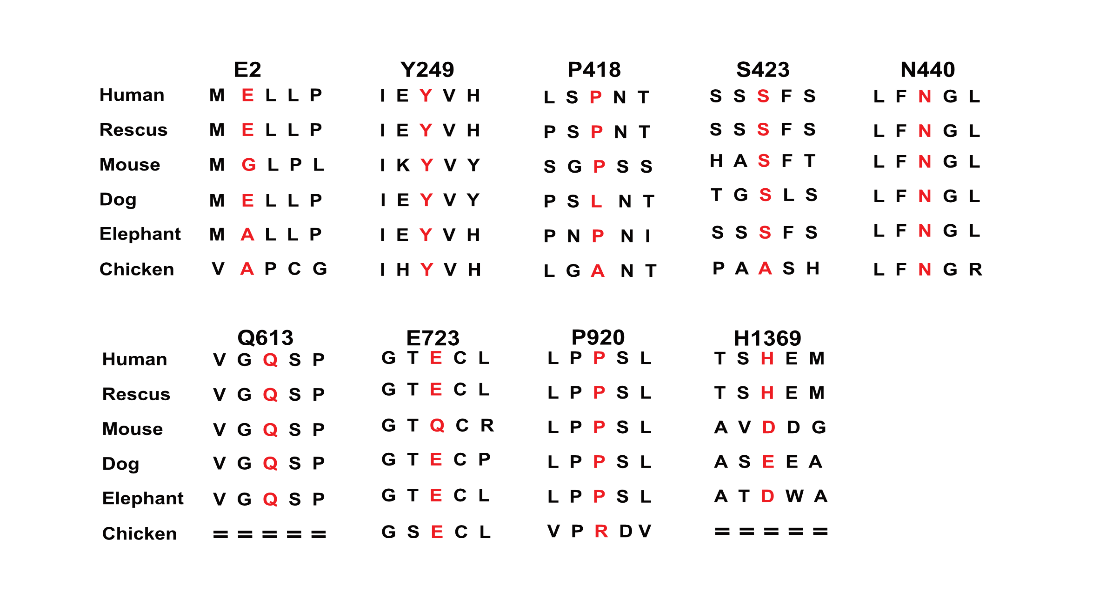


**Fig. S1 Conservation of rare variants in the *MST1R* gene identified in individuals with TOF**


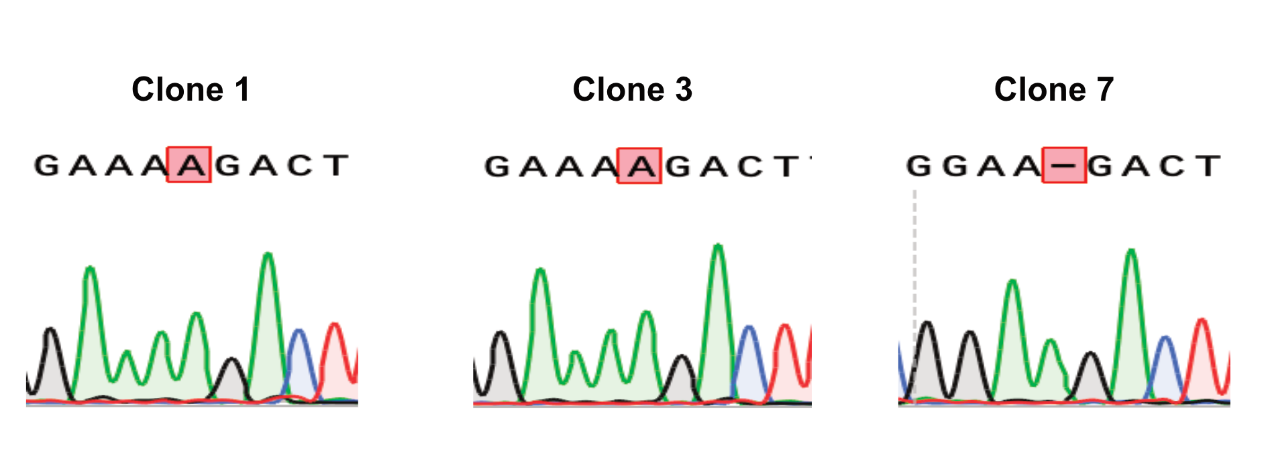


**Fig. S2 Sanger sequencing of positive *MST1R* knockout clones**

**
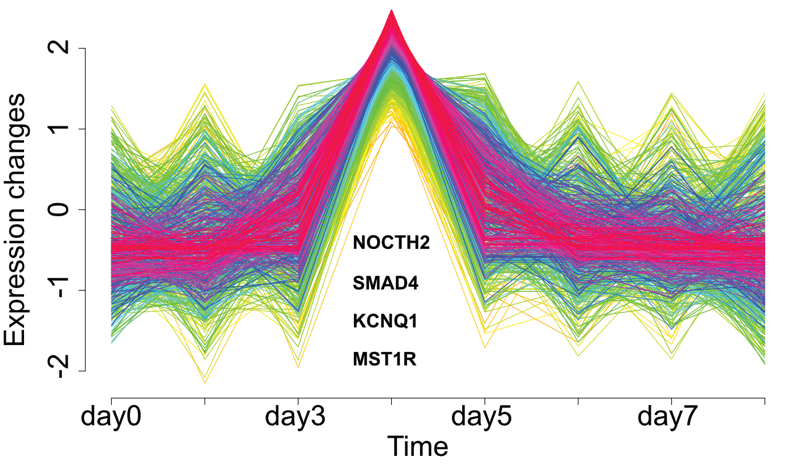
**

**Fig. S3 Mfuzz clustering of the time-course gene expression profiles**


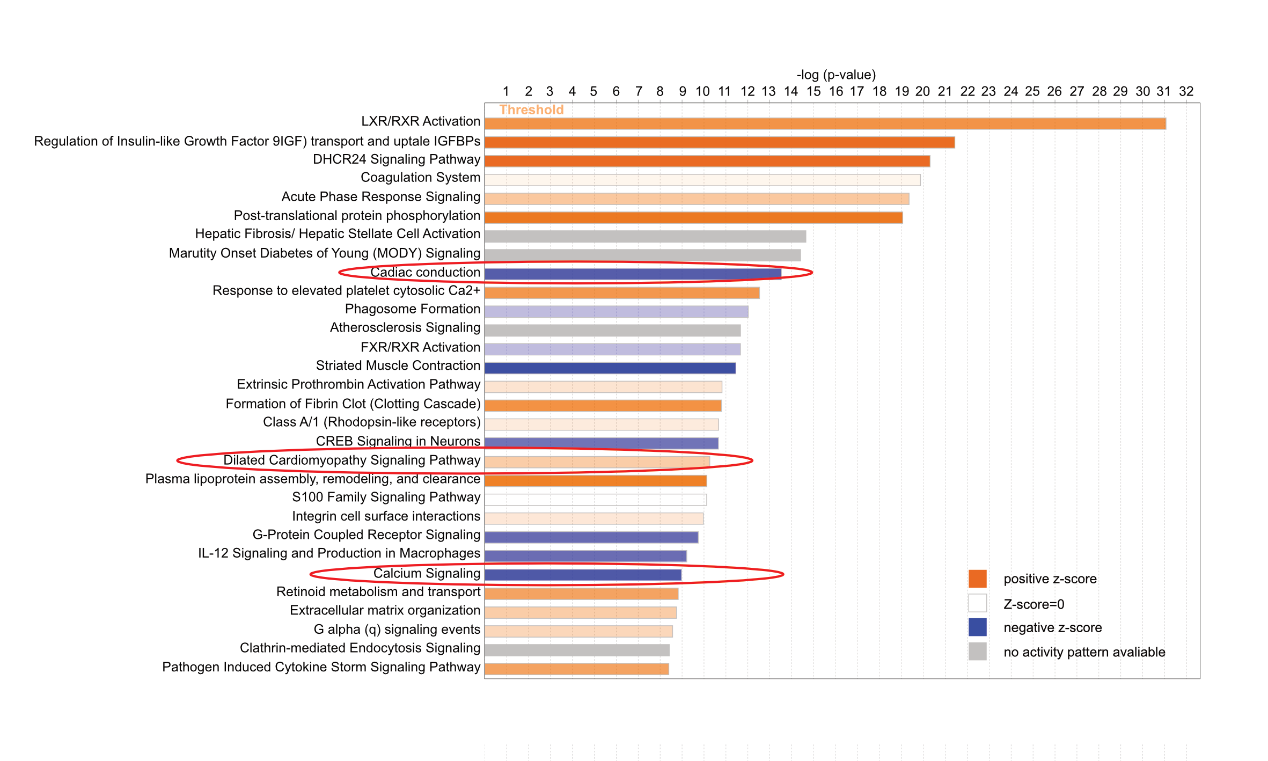


**Fig. S4 Top 30 canonical pathways identified by IPA analysis**

Red circles indicate pathways that are consistent with GO and KEGG analysis results


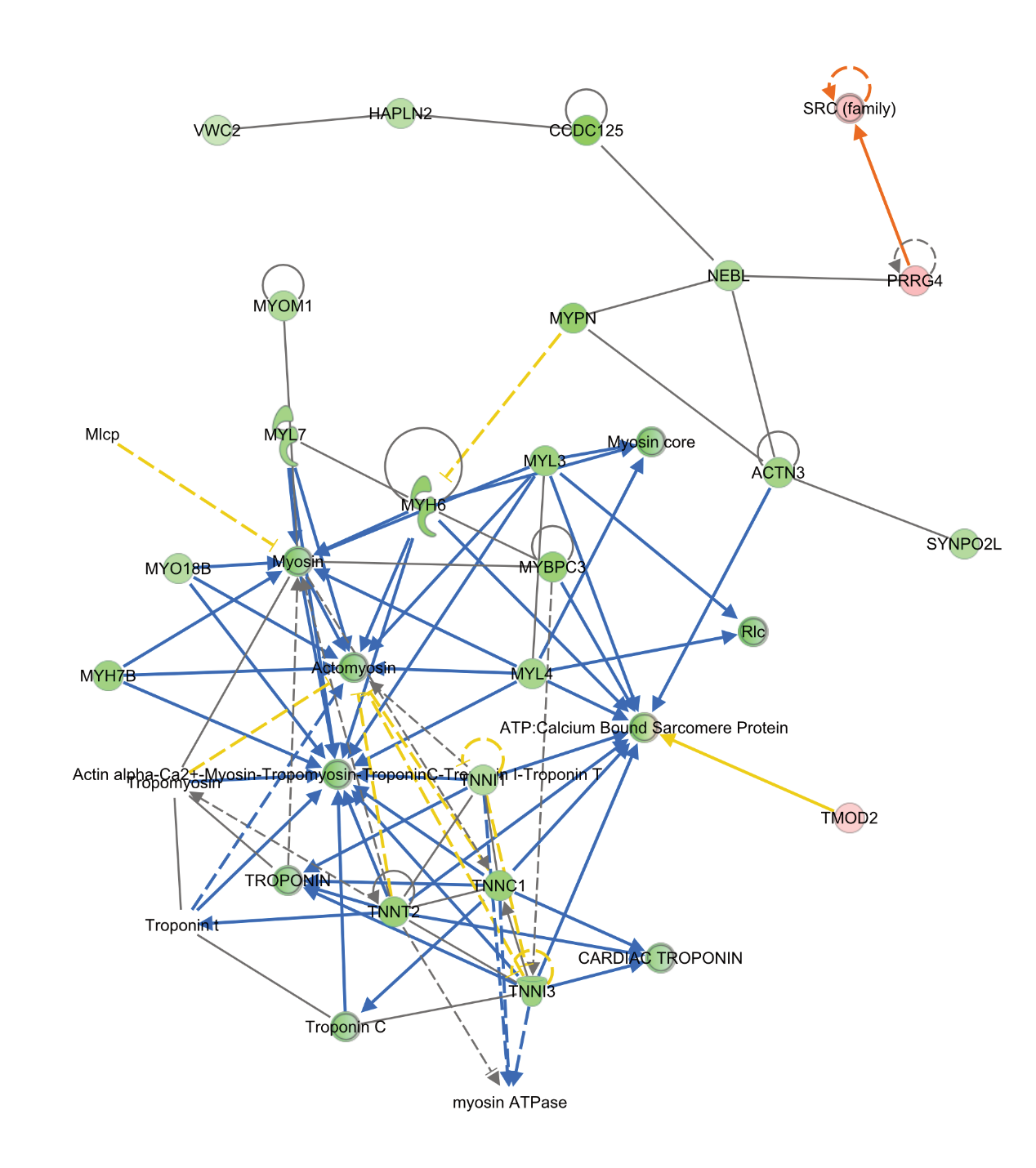


**Fig. S5 The core analysis of IPA identified core terms and the network interactions related to cardiovascular diseases**

The upregulated and downregulated genes are indicated as red and green, respectively
